# PRMT-1 and p120-Catenin as EMT Mediators in Osimertinib Resistance in NSCLC

**DOI:** 10.3390/cancers15133461

**Published:** 2023-07-01

**Authors:** Kavya Sri Racherla, Katrina Dovalovsky, Meet Patel, Emma Harper, Jacob Barnard, S M Nasifuzzaman, Mason Smith, Riya Sikand, Eva Drinka, Neelu Puri

**Affiliations:** 1 Department of Biomedical Sciences, University of Illinois College of Medicine at Rockford, Rockford, IL 61107, USA; 2 Department of Pathology, University of Wisconsin Health, Swedish American Hospital, Rockford, IL 61104, USA

**Keywords:** Osimertinib, NSCLC, epidermal growth factor receptor, EMT, p120-catenin, PRMT-1, reactive oxygen species (ROS)

## Abstract

**Simple Summary:**

Non-small-cell lung cancer (NSCLC) patients become resistant to targeted therapies such as tyrosine kinase inhibitors (TKIs) through mutations in the EGFR gene and by overexpression of proteins such as p120-catenin and PRMT-1. We studied EGFR wild-type and mutated EGFR in parental and Osimertinib-resistant cell lines. Our results showed overexpression of p120-catenin, PRMT-1, and Kaiso factor in Osimertinib-resistant cell lines compared to parental cell lines. We also found co-localization of PRMT-1 and Kaiso factor in resistant cell lines. Overexpression of p120-catenin and PRMT-1 was found in tumor samples recovered from smokers as compared to non-smokers. Further studies showed that inhibiting p120-catenin mediated increased Osimertinib efficiency and decreased wound healing compared to mock siRNA controls. These results indicate that p120-catenin and PRMT-1 could play a vital role in Osimertinib resistance and could be potential targets to increase TKI efficiency.

**Abstract:**

Osimertinib, an irreversible tyrosine kinase inhibitor, is a first-line therapy in EGFR-mutant NSCLC patients. Prolonged treatment with Osimertinib leads to resistance due to an acquired C797S mutation in the EGFR domain and other mechanisms, such as epithelial-mesenchymal transition (EMT). In this study, we investigated the role of PRMT-1 and p120-catenin in mediating Osimertinib resistance (OR) through EMT. These studies found upregulation of gene and protein expression of PRMT-1, p120-catenin and Kaiso factor. Knockdown of p120-catenin using siRNA increased OR efficacy by 45% as compared to cells treated with mock siRNA and OR. After 24 h of transfection, the percentage wound closure in cells transfected with p120-catenin siRNA was 26.2%. However, in mock siRNA-treated cells the wound closure was 7.4%, showing its involvement in EMT. We also found high levels of p120-catenin expressed in 30% of smokers as compared to 5.5% and 0% of non-smokers and quit-smokers (respectively) suggesting that smoking may influence p120-catenin expression in NSCLC patients. These results suggest that biomarkers such as PRMT-1 may mediate EMT by methylating Twist-1 and increasing p120-catenin expression, which causes transcriptional activation of genes associated with Kaiso factor to promote EMT in Osimertinib-resistant cells.

## 1. Introduction

In the United States, lung cancer accounts for about 21% of all cancer-related deaths [1]. In 2023, approximately 238,340 new cases and 127,070 deaths are expected to be due to lung cancer [1]. Non-small-cell lung cancer (NSCLC) makes up over 75% of lung cancer cases [2,3]. Patients with NSCLC are usually diagnosed at a very advanced stage and the prognosis of NSCLC is currently the lowest among all cancers with a 5-year survival rate of approximately 27% for all NSCLC stages [4]. Due to the low survival rates of NSCLC patients, many studies have focused on several molecular targets, such as epidermal growth factor receptor (EGFR) [5]. About 60% of NSCLC patients abnormally express EGFR, making it a potential therapeutic target for NSCLC [6]. EGFR belongs to the ErbB family of receptor tyrosine kinases and is amplified in NSCLC along with another tyrosine kinase receptor, c-Met. EGFR is stimulated by its extracellular ligand, which causes it to dimerize and activate its tyrosine kinase domain that further stimulates downstream oncogenic signaling cascades, such as the MAPK, PI3K/Akt and STAT pathways [7]. In 25% of NSCLC tumors, EGFR mutations have been observed and overexpression is commonly observed in 75% of these cases [8]. In more than 90% of patients with EGFR mutations, short in-frame deletions in exon 19 and point mutations in exon 21 (L858R) occur [7,8]. These mutations lead to constitutive activation of downstream signaling pathways, leading to cell proliferation and cancer progression [8]. Thus, the current therapy to treat NSCLC with activating EGFR mutations relies on tyrosine kinase inhibitors (TKIs) as an alternative to toxic chemotherapy [9].

Tyrosine kinase inhibitors such as Erlotinib and Gefitinib (first generation TKIs) are used as molecular targeted therapies and are used to treat patients harboring L858R activating mutations. However, patients often acquire a secondary mutation called the T790M mutation that makes them resistant to first-generation TKIs [10]. Third-generation TKIs such as Osimertinib bind to the cysteine 797 residue at the ATP binding site of EGFR. Osimertinib has been proven to provide superior progression-free survival [8] as compared to Erlotinib and Gefitinib, with a median progression free survival (PFS) of 18.9 months as compared to Gefitinib therapy with a PFS of 10.2 months [10]. However, resistance to Osimertinib develops after prolonged use due to multiple pathways, including C797S mutation, c-MET amplification and other alternate signaling pathways [11]. Epithelial to mesenchymal transition (EMT) is another process which plays an important role in tumor progression, metastasis and resistance to TKIs in various cancers, including NSCLC [11,12,13,14]. During EMT, epithelial cells obtain mesenchymal characteristics by losing their polarity and adhesion to gain migratory properties [11,12]. After cells have undergone EMT, they experience a loss of tight junction proteins, such as Claudin and E-cadherin, and increased expression of Twist, ZEB1, Snail, and Slug proteins that transcriptionally repress tight junction proteins [11,12]. Additionally, previous studies have found that cigarette smoke increases EMT [15,16,17].

Protein arginine methyltransferase I (PRMT-1) is a type I arginine methyltransferase which is responsible for approximately 75% of arginine methylation in cells [18]. The upregulation of PRMT-1 has been found to promote the proliferation and transformation of cancer cells in various cancers, including lung cancer, breast cancer and colon cancer [18]. Studies have also shown that PRMT-1 may mediate EMT by methylation of Twist and activation of ZEB1 [19,20,21]. The methylation of Twist by PRMT-1 downregulates E-cadherin and switches E-cadherin to N-cadherin, leading to translocation of p120-catenin and β-catenin from the plasma membrane to the cytoplasm, promoting EMT [19,22]. Thus, overexpression of PRMT-1 may play an important role in causing TKI resistance in NSCLC.

p120-catenin is a protein that stabilizes the epithelial adherens junction (AJ), which is an E-cadherin-based complex that maintains cell-cell junction integrity [23]. When E-cadherin is downregulated during cancer progression and EMT, p120-catenin translocates into the cytoplasm where it acts as a regulator of Rho GTPases (Rho A, Rac1 and cdc42), which are involved in EMT, cell migration, and growth factor signaling [23,24]. After translocation to the cytoplasm, p120-catenin shuttles inside the nucleus to interact with Kaiso factor, which is a transcriptional repressor of Wnt target genes [23,24]. The binding of p120-catenin with Kaiso factor causes downregulation of Wnt target genes, such as Snail, Slug and Twist, which leads to inhibition of the E-cadherin promoter, causing EMT [24,25].

Although several studies are dedicated to elucidating the role of EMT in cancer progression and tumorigenicity, the role of p120-catenin and PRMT-1 in TKI resistance is minimally studied. In this study, we investigated the role of PRMT-1 and p120-catenin in mediating resistance to Osimertinib in NSCLC. We found that expression of p120-catenin and PRMT-1 was upregulated in TKI-resistant cells and knockdown of p120-catenin increased efficacy of Osimertinib. In addition, we analyzed tumor sections from smokers and non-smokers and found that expression of p120-catenin was increased in smokers as compared to non-smokers. These studies indicate that p120-catenin and PRMT-1 may play a role in TKI resistance.

## 2. Materials and Methods

### 2.1. Tyrosine Kinase Inhibitors and Epidermal Growth Factor Ligands

Erlotinib hydrochloride was acquired from LC labs (Woburn, MA, USA) and dissolved in dimethyl sulfoxide (DMSO) at 20 mM. Aliquots of 20 µL were stored at −20°. ZM 323881 hydrochloride (5-((7-Benzyloxyquinazolin-4-yl)amino)-4-fluoro-2-methylphenol hydrochloride) was purchased from Tocris Bioscience (Minneapolis, MN, USA), dissolved in DMSO at 1 mM, after which aliquots were stored at −20 °C. Epidermal growth factor (EGF) was purchased from Peprotech (Rocky Hill, NJ, USA), resuspended in PBS at a 15 ng/µL concentration, and stored at −20 °C.

### 2.2. Antibodies

Rabbit monoclonal antibodies for E-cadherin, Claudin, and Vimentin were purchased from Cell Signaling Technologies (Danvers, MA, USA) as a part of the EMT antibody sampler kit (Cat. No. 9782). Rabbit monoclonal PRMT-1 antibody was purchased from Cell Signaling Technologies (Cat. No. 2449S), mouse monoclonal p120-catenin/Delta catenin antibody was purchased from Invitrogen (Waltham, MA, USA, Cat No. 33-9700). Rabbit polyclonal antibody against Kaiso factor was used for immunofluorescence (H-154) (Cat. No. sc-98589) and mouse monoclonal antibody against Kaiso factor was used for immunoblotting (D-10) (Cat. No. sc-365428) and were both purchased from Santa Cruz Biotechnology (Dallas, TX, USA). Mouse monoclonal antibody for β-Actin (Cat. No. A5441) was purchased from Sigma Aldrich (St. Louis, MO, USA). Anti-rabbit IgG (Cat. No. 31460) and anti-mouse IgG (Cat. No. A16078) secondary monoclonal antibodies were obtained from Invitrogen. All the primary antibodies were diluted to 1:1000 in 1% BSA made in 1X TBST. The secondary antibodies were made at 1:10,000 dilution with 1% blocking grade milk (Cat. No. 170-6404XTU) from Bio-Rad (Hercules, CA, USA).

### 2.3. Cell Lines and Tissue Culture Techniques

The H358, H2170, H3255 and H1975 cell lines were cultured with 5% CO_2_ at 37 °C following the protocol of the American Type Culture Collection (ATCC). The H2170 and H358 cell lines have EGFR wild-type status, while H1975 has two EGFR tyrosine kinase domain mutations—L858R and T790M [26,27]. H3255 has a single EGFR tyrosine kinase domain mutation-L858R [27,28]. All four cell lines were cultured in Roswell Park Memorial Institute Medium (RPMI 1640) (Thermo Fisher Scientific, Pittsburg, PA, USA). The H3255ER and H1975ER lines were cultured in media supplemented with 100 nM and 10 µM Erlotinib, respectively, and the H2170OR, H358OR, H3255OR and H1975OR lines were cultured in media supplemented with 5 µM, 10 µM, 24 nM and 1 µM Osimertinib, respectively.

### 2.4. Primers for qPCR

The primers for qPCR experiments were purchased from Integrated DNA Technologies (IDT, Coralville, IA, USA), as seen in Table 1. For qPCR, 1 × 10^6^ cells were plated in a 60 mm dish and were given serum-free media (RPMI + 0.5% BSA) for 24 h. The following day, RNA was extracted using Trizol. The RNA samples were quantified and converted to cDNA (Applied Biosystems, Waltham, MA, USA). The cDNA was then used to perform qPCR using PowerUp SYBR green from Applied Biosystems. Furthermore, 2^(−Δ(ΔCt))^ was calculated to evaluate the fold changes in gene expression or mRNA levels of proteins of interest.

### 2.5. Immunoblotting

A total of 3.5 × 10^5^ cells were seeded in 35 mm petri-dishes and were allowed to grow for 24–48 h or until they reached 90% confluency. The cells were then serum-starved (RPMI with 0.5% BSA) for 24 h. H2170OR, H358OR, H3255OR and H1975OR cell lines were then treated with 5 µM/10 µM/24 nM/1 µM Osimertinib, respectively. H2170ER, H358ER and H1975ER were treated with 10 µM Erlotinib and H3255ER was treated with 100 nM Erlotinib in serum-free media for 24 h. The cells were then treated with EGF ligand (15 ng/mL in serum-free media) for 2.5 min before lysate collection. The cell lysate was then produced, electrophoresed and transferred on the nitrocellulose membrane using a semi-dry transfer method [26]. The membrane was then blocked and probed with primary antibody overnight at 4 °C. The next day, a suitable secondary antibody was applied to the membranes, after which they were developed using a Pierce ECL chemiluminescence substrate (Thermo Fisher Scientific, Waltham, MA, USA). The developed films were then scanned for quantitative densitometric analysis using Image J software (version 1.46r).

### 2.6. Immunofluorescence

NSCLC cells were plated in an 8-well chamber slide and the procedure was performed as described earlier [12]. On the first day, cells were fixed, permeabilized, blocked and were incubated with a mixture of two primary antibodies (1:400) prepared in 1% BSA for double-staining of p120-catenin/Kaiso factor. The following day, cells were incubated with anti-mouse/anti-rabbit IgG secondary antibodies conjugated with DyLightTM 488 [1:250] or Cy3 for 1 h in the dark. After incubation with secondary antibodies, cells were incubated with Hoescht dye [1:2500] for nuclear staining for 15 min. The stained images were captured using an Olympus Fv10i Fluoview confocal microscope. The average intensity of fluorescence was quantitatively measured using the Olympus Fluoview image analysis software (version 04. 02) and the fold change was calculated. The percentage colocalization of two proteins inside the nucleus was determined using the JacoP Plugin of ImageJ software (version 1.46r).

### 2.7. SiRNA Transfection

Cells were seeded a day before the transfection. On the day of transfection, control siRNA or p120-catenin-siRNA were diluted in Opti-MEM media along with the transfection reagent Dharmafect-2 (Cambridge, UK, Horizon Discovery). The transfection was performed as per the manufacturer’s protocol as described earlier [21]. The cell lysates were collected after 24, 48 and 72 h for the Western blotting experiment.

### 2.8. MTT Cell Viability Assay

NSCLC cells were seeded as 5500 cells/well in a 96-well plate and were allowed to grow until 60% confluency in antibiotic-free RPMI media. The cells were then transfected with either the mock siRNA or p120-catenin siRNA for 24 h, followed by 10 µM Osimertinib for 24 h. MTT viability assay was performed as described earlier [29]. Each treatment condition was conducted in six total replicates.

### 2.9. Wound-Healing Assay

A total of 3.5 × 10^5^ cells/well were seeded in a 6-well plate and were allowed to grow until 80% confluency in antibiotic-free RPMI media. The cells were then transfected with either the mock siRNA or p120-catenin siRNA for 24 h. The transfection media was removed, and a wound/scratch was created using a 200 µL tip; the wound/scratch was treated with 10 µM Osimertinib-containing media and incubated for 24 h at 37 °C. The images were taken at 40× magnification at time zero and after 24 h of Osimertinib treatment. The experiment was performed in triplicates and the images were analyzed using T-scratch software (version 1.0).

### 2.10. Immunohistochemistry

Tissue slides of NSCLC tumors fixed in formalin were obtained. The tissue slides were double-stained or single-stained depending on the biomarker being identified. We performed double-staining of p120-catenin and PRMT-1 and examined the staining of each biomarker individually. All cases were retrieved from the pathology archives at the OSF St. Anthony’s and Swedish American Hospital according to an approved Institutional Review Board protocol (351597-11 and 1 March 2022). Immunostaining protocols were carried out following the previously described procedures [30]. Negative controls for immunostaining were prepared by replacing the primary antibody with nonimmune rabbit serum. The statistical analysis was performed using chi square analysis and the *p*-value was calculated using Fisher’s exact test to determine the correlation between the PRMT-1/p120-catenin biomarker expression and the smoking status of NSCLC patients.

## 3. Results

### 3.1. Increased Gene Expression of EMT-Related Proteins in Osimertinib-Resistant Wild-Type and Mutant EGFR NSCLC Cells

**Figure 1:**
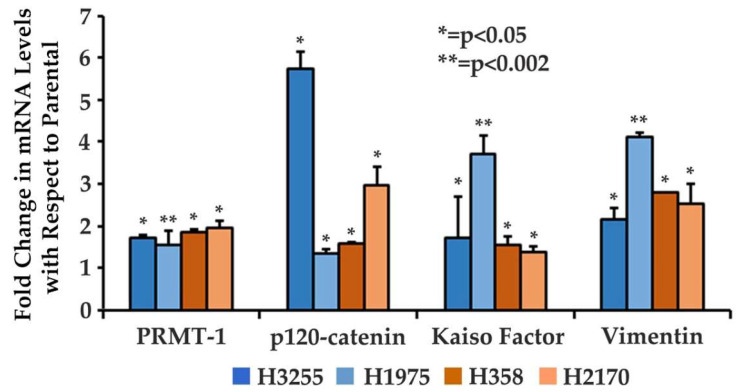
Modulation in gene expression of EMT biomarkers in wild-type and EGFR mutant Osimertinib-resistant cell lines compared to parental cells using qPCR. A total of 3.5 × 10^5^ cells were seeded in 35 mm dishes and allowed to grow for 24 h, following which cells were kept in serum-free medium for 24 h, after which total mRNA was isolated. The mRNA was quantified, and cDNA was converted. We further performed qPCR to study the gene expression of EMT biomarkers. The fold changes were calculated using the 2^(−ΔΔCt)^ method and the data was normalized with GAPDH; parental cells were considered to have a fold change of 1. The results were statistically significant with respect to parental cells by Dunnett’s test for H2170OR, H358OR, H3255OR (n = 2, *p* < 0.05) and H1975OR cells (n = 2, *p* < 0.002).

We analyzed modulation in the gene expression of EMT-related proteins at the mRNA level in NSCLC cell lines. We performed qPCR to analyze gene expression in the Osimertinib-resistant wild-type EGFR cell lines, H358OR and H2170OR. The real-time PCR results showed that there was an increase in the gene expression of PRMT-1, p120-catenin, Kaiso factor and Vimentin by 1.8-fold, 1.6-fold, 1.6-fold and 2.8-fold, respectively, in the H358OR cells compared to the parental cells, and by 2-fold, 3-fold, 1.4-fold and 2.3-fold, respectively, in the H2170OR cells compared to the parental cells (Figure 1). We also performed a qPCR in the Osimertinib-resistant EGFR mutant cell lines H3255OR and H1975OR compared to parental cells. We observed that there was an increase in the gene expression of PRMT-1, p120-catenin, Kaiso factor and Vimentin by 1.7-fold, 5.7-fold, 1.7-fold and 2.2-fold, respectively, as compared to the H3255P, and there was increased fold difference observed for PRMT-1, p120-catenin, Kaiso factor and Vimentin by 1.5-fold, 1.3-fold, 3.3-fold and 4.1-fold in H1975OR, respectively, as compared to the H1975P cells (Figure 1).

### 3.2. Increased Expression of Key Protein Biomarkers of the EMT Pathway in H358OR and H1975OR NSCLC Cell Lines

**Figure 2:**
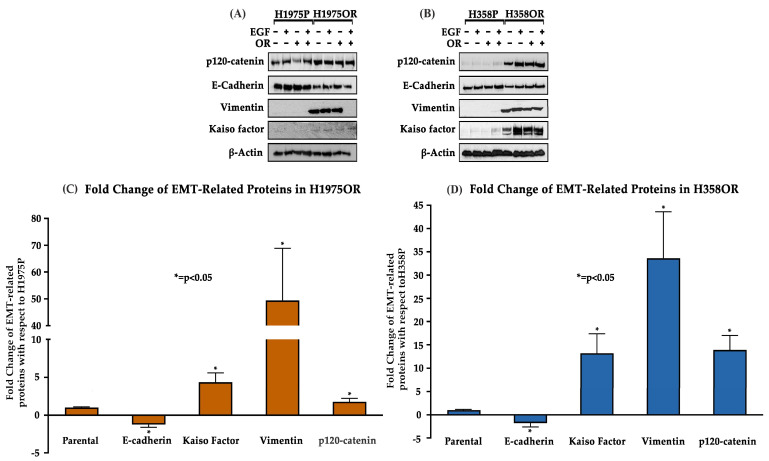
Modulation of EMT-related proteins in the TKI resistant EGFR wild-type H358 and EGFR-mutant H1975 NSCLC cells. (**A**,**B**) 3.5 × 10^5^ cells were seeded in a 35 mm dish and allowed to grow for 24–48 h until they reached 90% confluency. The cells were then serum-starved using serum-free media (plain media with 0.5% BSA) for 24 h and then treated with EGF (15 ng/mL for 2.5 min) and/or Osimertinib for 24 h. (**C**,**D**). The modulations in the protein expression were calculated with respect to parental cells by densitometric analysis using the ImageJ software (version 1.46r); the results were statistically significant with respect to parental cells by the Dunnett’s test (n = 2, *p* < 0.05).

For elucidating the mode of resistance to Osimertinib in the EGFR-wild type H358 and EGFR-mutant H1975 NSCLC cell lines, immunoblotting was performed to determine the varying levels of EMT-related proteins. We compared their expression in the parental cell lines to their expression in the resistant cells that were subjected to different treatment conditions: diluent (no treatment), EGF ligand treatment, Osimertinib treatment and both EGF and OR treatment. The analysis of our results showed that there was downregulation of E-cadherin by 1.8-fold and upregulation of the mesenchymal marker, Vimentin, by 34-fold in H358OR as compared to the H358P. Similarly, the EMT markers’ Kaiso factor and p120-catenin were found to be upregulated by about 13-fold and 14-fold, respectively, in H358OR as compared to the H358P cells (Figure 2, Supplementary Materials, Table S4).

Previous studies in our lab have shown downregulation of E-cadherin, an adherens junction protein, in H2170ER and H358ER as compared to the parental cell lines [12]. Hence, we were interested in comparing changes in expression of several EMT-associated proteins in H1975OR cells. Vimentin, p120-catenin and Kaiso factor were upregulated by 48-fold, 1.77-fold and 4.36-fold, respectively, in the H1975OR cells compared to the H1975P, while E-cadherin was downregulated by 1.3-fold (Figure 2,  Supplementary Materials, Table S5). 

### 3.3. Colocalization of p120-Catenin and Kaiso Factor in Erlotinib- and Osimertinib-Resistant H3255 NSCLC Cells

**Figure 3:**
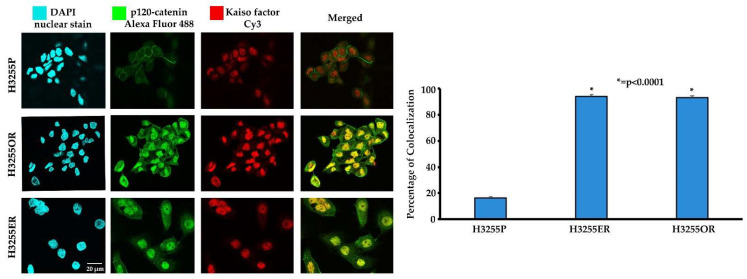
Colocalization of p120-catenin and Kaiso factor in TKI-resistant H3255ER and H3255OR NSCLC cells. A total of 20,000 cells were plated per well in an 8-well chamber slide. The cells were fixed, permeabilized and probed with p120-catenin and Kaiso factor antibodies. DAPI was used as a nuclear stain, Alexa Fluor 488 conjugated secondary antibody was used to detect p120-catenin and Cy3 conjugated secondary antibody was used to detect Kaiso factor. Images were captured using an Olympus fv10i confocal microscope and colocalization was analyzed using the ImageJ (version 1.46r, JacoP Plugin) software. The results were statistically significant with respect to parental cells by two-tailed *t*-test analysis for n = 3.

The translocation of p120-catenin into the nucleus due to EMT causes its interaction with the Kaiso factor and this interaction activates the Wnt pathway genes, promoting EMT and invasiveness [23,24,25,31,32]. We performed an immunofluorescence double-staining experiment with anti-p120-catenin primary antibody detected with Alexa 488 fluorophore-conjugated mouse secondary antibody and anti-Kaiso factor primary antibody detected using Cy3 fluorophore-conjugated rabbit secondary antibody to study the interaction of both the proteins. Colocalization of p120-catenin and Kaiso factor was captured using an Olympus fv10i confocal microscope and quantified using ImageJ software (version 1.46r, JacoP Plugin). The results indicated a Mander’s overlap coefficient of about 0.95 or 95% colocalization in H3255ER and 0.94 or 94% colocalization in H3255OR as compared to 0.16 or 16% colocalization in H3255P (Figure 3).

### 3.4. Increased Nuclear Fluorescence of PRMT-1 in the Erlotinib- and Osimertinib-Resistant H3255 and H1975 NSCLC Cells

**Figure 4:**
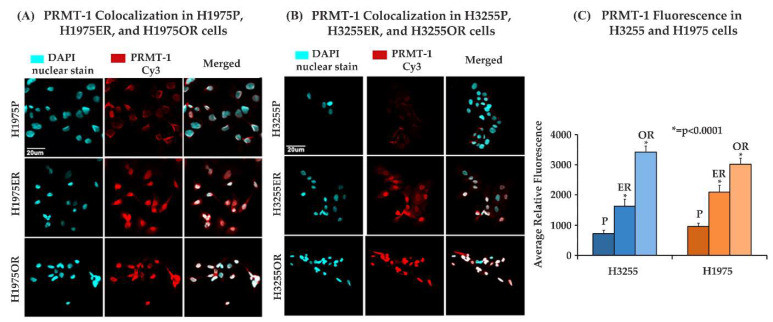
Increase in nuclear fluorescence of PRMT-1 in the TKI-resistant H1975 and H3255 cells. (**A**,**B**). 20,000 cells per well were plated in an 8-well chamber slide. The cells were fixed, permeabilized and probed for PRMT-1. DAPI was used as a nuclear stain while Cy3 conjugated rabbit secondary antibody was used for detection of PRMT-1. An Olympus fv10i microscope was used to capture images. (**C**) Fluorescence was quantified using Olympus FluoView image software (version 04. 02) on parental (P), Erlotinib resistant (ER) and Osimertinib resistant (OR) cells. The results were statistically significant with respect to parental cells by two-tailed *t*-test analysis (n = 3, *p* < 0.0001).

PRMT-1 is a regulator of EMT that increases the invasive and migratory properties, which promotes lung cancer progression and metastasis. PRMT-1 promotes EMT by epigenetic methylation of Twist-1, a transcription factor and a repressor of E-cadherin [19]. We performed an immunofluorescence experiment with the anti-PRMT-1 primary antibody detected using Cy3 fluorophore-conjugated rabbit secondary antibody. The fluorescence images were captured using an Olympus fv10i confocal microscope and analyzed using Olympus FluoView image analysis software (version 04. 02). We found that the average fluorescence intensity of PRMT-1 was upregulated by 2.2-fold and 3.2-fold in H1975ER and H1975OR cells, respectively, as compared to the H1975P cells. PRMT-1 was also found to be upregulated by 2.3-fold and 4.8-fold in the H3255ER and H3255OR cells, respectively, as compared to the H3255P cells (Figure 4).

### 3.5. Effect of p120-Catenin Knockdown on Reversal of EMT and Osimertinib Resistance in H358OR Cells

**Figure 5:**
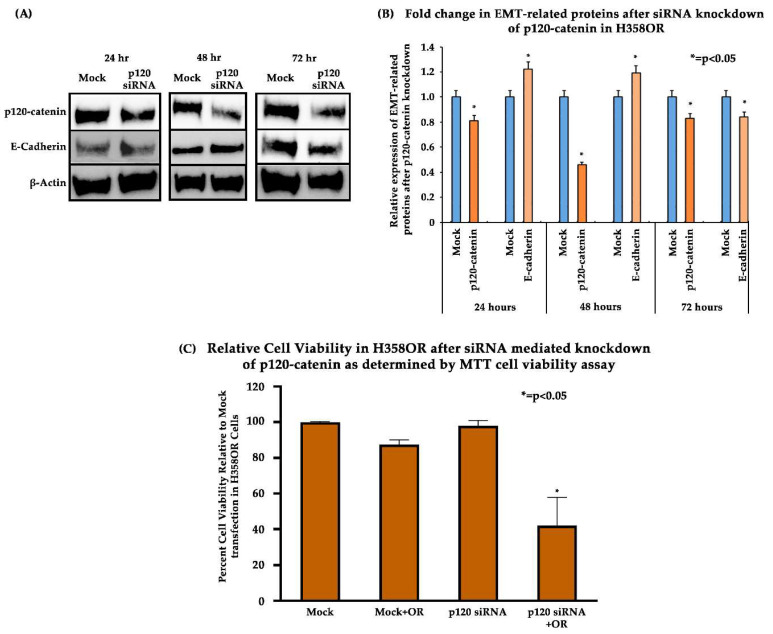
Effect of p120-catenin knockdown on EMT and Osimertinib resistance in H358OR cells by immunoblotting. (**A**) A total of 3.5 × 10^5^ cells/well were seeded in a 6-well plate in antibiotic-free media until they achieved 80% confluency. The cells were then treated with either mock or p120-catenin siRNA and lysates were collected after 24, 48 and 72 h to perform immunoblotting. (**B**) The graph represents the fold change after immunoblotting of EMT-related proteins (p120-catenin and E-cadherin) after siRNA knockdown of p120-catenin. The modulations in the protein expression were calculated by densitometric analysis with respect to the parental cell lines using the ImageJ software and the results were statistically significant by two-tailed *t*-test analysis (n = 2, *p* < 0.05). (**C**) A total of 5500 cells/well were seeded in a 96-well plate in antibiotic-free media a day before transfection. The cells were then transfected with either mock or p120-catenin siRNA for 24 h and further treated with Osimertinib (10 µM) for 24 h. The percentage cell viability was analyzed with respect to mock siRNA transfection and treatment with Osimertinib compared to the combination of p120 siRNA with Osimertinib treatment and results were found to be statistically significant by two-tailed *t*-test analysis in both comparisons (n = 2, *p* < 0.05).

The Osimertinib-resistant H358OR cell line possesses a KRAS mutation that mediates Osimertinib resistance and, because the expression of p120-catenin was found to be upregulated in them, we wanted to investigate if the knockdown, or inhibition of p120-catenin in the H358OR cells, would reverse the process of EMT. Thus, we targeted p120-catenin mRNAs using siRNA in the H358OR cells for 24, 48 and 72 h and then performed immunoblotting. The results of immunoblotting indicated that, after 24 h of transfection, there was about 19% downregulation of the p120-catenin protein and 20% upregulation of the E-cadherin protein. After 48 h of transfection, we observed a 55% downregulation of p120 catenin, while there was an increase in E-cadherin expression by 18% (Figure 5A,B). We then performed an MTT assay to study the effect of p120-catenin downregulation on the efficacy of Osimertinib. The results indicated that, after knockdown of p120-catenin, the efficacy of Osimertinib was increased by about 45% as compared to the mock siRNA-transfected cells and the Osimertinib-treated cells. The percentage viability of cells with different treatments was calculated with respect to the viability of the mock siRNA-transfected cells (Figure 5C, Supplementary Materials, Table S6). Thus, these results suggest that targeting p120-catenin by knockdown has the potential to reverse EMT characteristics in the H358OR cell lines.

### 3.6. Effect of p120-Catenin Knockdown and Osimertinib Treatment on EMT and Osimertinib Efficacy in H358OR Cells Using a Wound-Healing Assay

**Figure 6:**
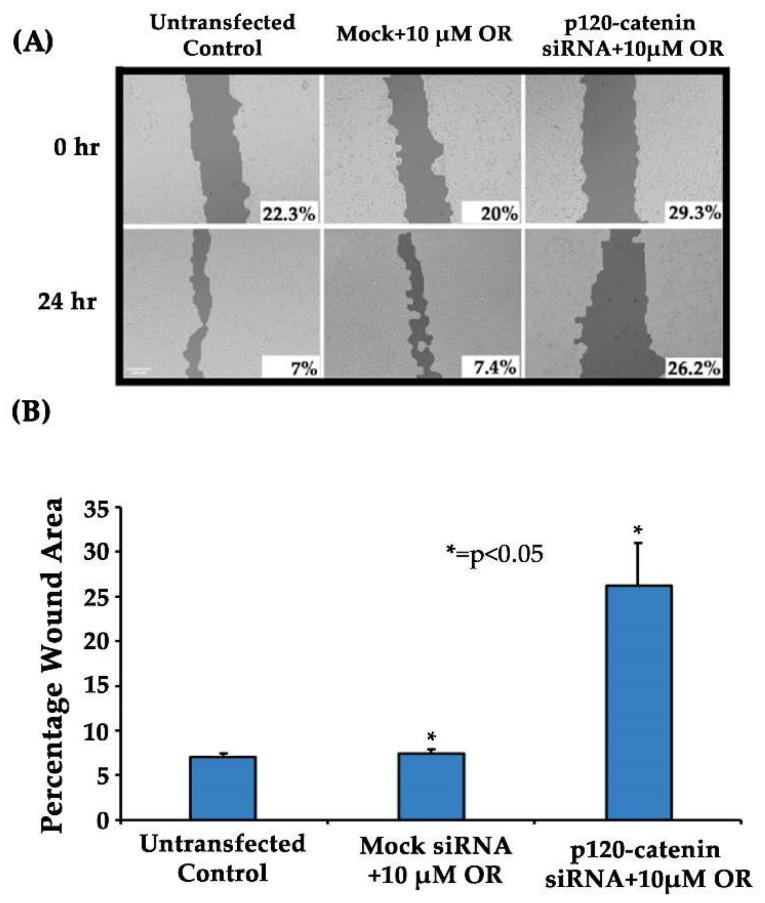
Effect of p120-catenin knockdown and Osimertinib treatment on EMT and Osimertinib resistance in H358OR cells by wound-healing assay. (**A**) A total of 3.5 × 10^5^ cells were seeded in antibiotic-free media in a 6-well plate in duplicates a day before transfection. The cells were treated with either mock or p120-catenin siRNA for 24 h. The transfection medium was removed after 24 h and a scratch was created using a 200 µL tip and then the cells were treated with Osimertinib (10 µM) for 24 h. The images were then captured at 0 h and 24 h time points after drug treatment. (**B**) The percentage wound area was plotted for the untransfected control (with no drug), mock siRNA-treated cells and p120-catenin-treated cells. The results were analyzed using T scratch software and the results were statistically significant with respect to the mock control by two-tailed *t*-test analysis (n = 3, *p* < 0.05).

We performed a wound-healing assay to study the cell migratory properties in the H358OR cells after knockdown of p120-catenin using siRNA for 24 h, following which we created a scratch and then treated the cells with Osimertinib for 24 h. The results indicated that there was slower migration in cells after p120-catenin knockdown as compared to the mock siRNA-transfected cells in the presence of the drug Osimertinib. The wound-closure percentage in p120-catenin siRNA-transfected cells was determined to be 26.2% after 24 h. The wound-closure percentage in mock siRNA-transfected cells was comparatively smaller at 7.4% (Figure 6). These results suggest that targeting p120-catenin through siRNA knockdown can reverse EMT or the migratory characteristics of the H358OR cells and potentially reverse Osimertinib resistance.

### 3.7. Expression of EMT-Related Biomarkers p120-Catenin/PRMT-1 in Lung Cancer Patient Tissues at Different Stages (Stage I–IV) and Correlation with the Smoking Status of Patients

**Figure 7:**
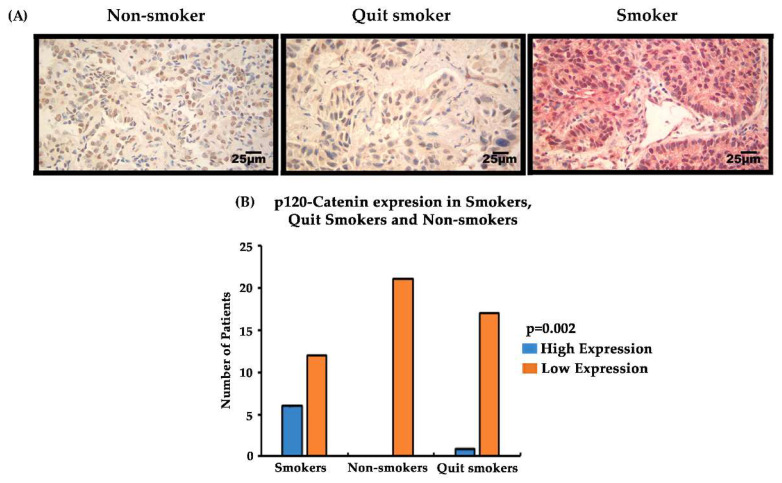
Expression of p120-catenin/PRMT-1 in lung cancer tissue sections from smokers, non-smokers, and quit-smokers. (**A**) Detection of PRMT-1 and p120-catenin in the NSCLC tumor sections via IHC were stained for PRMT-1 (brown) and p120-catenin (pink/red). PRMT-1 was mainly detected in the nucleus and p120-catenin in the cytoplasm and occasionally in the nucleus. Images were taken at 40× magnification. (**B**) Graphical representation of high and low expression of p120-catenin in the smoker, non-smoker and quit-smoker NSCLC patients, respectively.

Cigarette smoke has been shown to induce EMT in patients and is also associated with lung cancer progression, metastasis, and drug resistance [33]. Since, in our study, the EMT-related biomarkers PRMT-1 and p120-catenin were found to be upregulated in the Osimertinib-resistant NSCLC cells, we performed a translational study with these biomarkers in the tumor samples of the NSCLC patients and correlated their expression with the smoking status of the patients. A total of 62 NSCLC tumor sections were stained (22 quit-smokers (quit at least 5 years ago), 20 smokers (smoked ≥30 pack-years) and 21 non-smokers) (Supplementary Materials, Table S1). The results indicated a higher expression of p120-catenin in smokers compared to quit-smokers and non-smokers. The statistical test was calculated using Fisher’s exact test to determine if smokers had higher expression of p120-catenin. A total of 30% of smokers had high expression of p120-catenin, compared to 0% in quit-smokers and 5.5% in non-smokers. The statistical analysis showed that the high expression of p120-catenin occurred most frequently in patients who were smokers as compared to the quit-smokers and non-smokers (*p* = 0.002, Supplementary Materials, Table S2 and Figure 7B). There was no significant correlation observed between PRMT-1 expression and smoking status of the patients (Supplementary Materials, Table S3).

## 4. Discussion

In our study, we analyzed the association between TKI-resistant NSCLC cell lines and EMT to gain a better understanding of the mechanism of resistance. ER/OR-resistant NSCLC cell lines were shown to exhibit EMT. Hence, we explored the role of potential biomarkers involved in EMT and demonstrated that E-cadherin was downregulated in ER/OR TKI-resistant cell lines compared to parental cell lines, with an upregulation of EMT promoting biomarkers, such as Vimentin, Kaiso factor, PRMT-1 and p120-catenin (Figure 1 and Figure 2). Additionally, our study revealed colocalization of p120-catenin, Kaiso factor, and PRMT-1 in the nucleus of TKI-resistant cells compared to their respective parental cell lines, suggesting activation of their respective signaling pathways that promoted EMT transition (Figure 3 and Figure 4). Due to these findings, we further analyzed the relationship between p120-catenin and TKI resistance with knockdown of p120-catenin, which resulted in an increase in the efficiency and effectiveness of Osimertinib (Figure 5). The results showed that effective knockdown of p120-catenin increased E-cadherin expression, decreased metastatic ability and increased efficacy of OR in the presence of p120-catenin siRNA (Figure 5 and Figure 6), indicating that p120-catenin could overcome TKI resistance and potentially reverse EMT. We also observed increased expression of p120-catenin in smokers compared to non-smokers and quit-smokers (Figure 7).

We began our study by analyzing the expression of biomarkers PRMT-1, p120-catenin, and Kaiso factor in Osimertinib-resistant NSCLC cells with mutant EGFR (H1975 and H3255) and wild-type EGFR (H358 and H2170) and compared them to their parental cell lines. Immunoblotting revealed downregulation of E-cadherin in H358ER, H358OR, H1975ER and H1975OR cells compared to the parental cells. In addition, PRMT-1, p120-catenin, Kaiso factor, and Vimentin were upregulated in H358ER and H358OR wild-type cell lines, as well as H358OR, H2170OR and H1975OR, compared to parental cell lines (Figure 2). Thus, high expression of these biomarkers confirmed the presence of EMT in the NSCLC lines. This is consistent with previous reports highlighting the overexpression of EMT proteins in Osimertinib-resistant cells [34,35]. A prior study suggested that overexpression of Twist-1 in EGFR-mutated NSCLC cells was associated with Osimertinib resistance and that inhibition of Twist-1 could be a novel strategy to overcome Osimertinib resistance [4,34,35]. Another study suggested that Osimertinib-resistant cells showed high expression of EMT-TF Zeb-1 and the use of dual histone deacetylase (HDAC) and hydroxy-3-methylglutaryl co-enzyme-A reductase (HMGR) inhibitors restored sensitivity of the cells to Osimertinib and reversal of EMT in vitro [35]. Hence, we further explored the role of PRMT-1, p120-catenin, and Kaiso factor in Osimertinib-resistant NSCLC cells.

Since p120-catenin and Kaiso factor were upregulated in Osimertinib-resistant NSCLC cells (Figure 2), we assessed their localization in the Osimertinib-resistant H3255 cell lines. p120-catenin is an adherens junction protein that stabilizes E-cadherin on the cell membrane. When E-cadherin is lost during EMT, p120-catenin translocates into the cytoplasm and eventually into the nucleus. Nuclear p120-catenin binds to Kaiso factor, which is a repressor of Wnt target genes (Snail, Slug, Twist-1). The binding of p120-catenin to Kaiso factor inhibits the repression of downstream Wnt pathway genes, thus leading to their activation [23]. Immunofluorescence of p120-catenin and Kaiso factor demonstrated nuclear colocalization in H3255ER and H3255OR cells, which indicates that they play a role in EMT (Figure 3). This finding complements previous studies by our lab which have shown nuclear colocalization of p120-catenin and Kaiso factor in the nucleus of H1975 and H2170 Erlotinib-resistant cells, indicating their role in Erlotinib resistance as well [21]. Similarly, the nuclear colocalization of p120-catenin and Kaiso factor in Osimertinib-resistant cells enable the two to activate the downstream Wnt pathway, which increases EMT [21].

**Figure 8:**
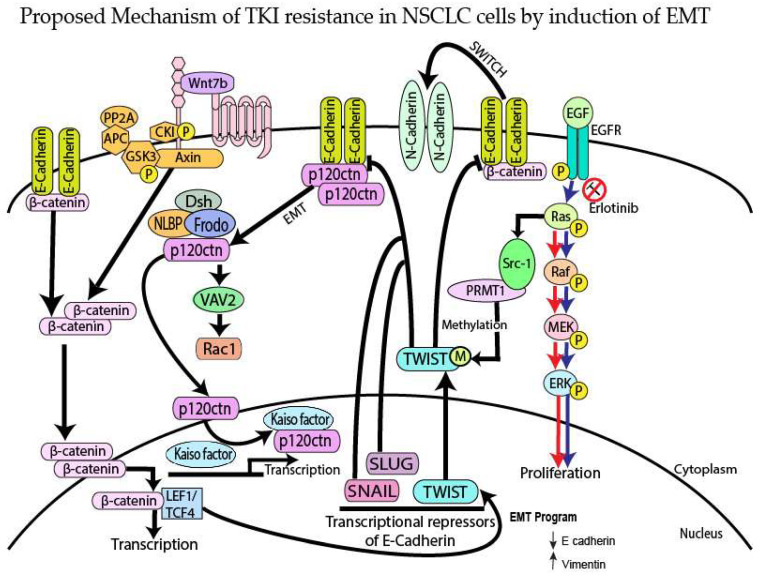
Proposed mechanism of TKI resistance in NSCLC cells by induction of EMT. NSCLC cells may undergo EMT due to two key molecules: PRMT-1 and p120-catenin. PRMT-1 is an enzyme that methylates Twist-1, a repressor of E-cadherin that is involved in the maintenance of epithelial phenotype. p120-catenin is involved in stabilizing E-cadherin on the plasma membrane, which acts as a de-repressor of Kaiso factor, a transcriptional factor of Snail, Slug and Twist.

Overexpression of PRMT-1 in bronchial epithelial cells leads to induction of EMT through a switch from E-cadherin to N-cadherin (Figure 8) [25]. Analysis of PRMT-1 localization in Osimertinib-resistant cells was performed since upregulation of PRMT-1 in Erlotinib-resistant cell lines was observed in our earlier studies. Additionally, previous studies from our lab have shown that inhibition of PRMT-1 leads to reversal of EMT and Erlotinib resistance in H358ER cell lines [21]. The immunofluorescence results in our study showed that PRMT-1 was mainly localized in the nucleus and was upregulated in the resistant cell lines compared to parental cell lines and may induce EMT and potential drug resistance in the NSCLC cell lines H1975ER/OR and H3255ER/OR (Figure 4).

Next, we examined the ability of p120-catenin knockdown to overcome Osimertinib resistance in H358OR cells (Figure 5 and Figure 6). Knockdown of p120-catenin was performed and resulted in increased E-cadherin expression, suggesting its potential to reverse EMT. Furthermore, an MTT assay was performed and showed increased Osimertinib sensitivity in the p120-catenin knockdown cells compared to the mock siRNA- and Osimertinib-treated cells. Thus, targeting p120-catenin may restore sensitivity to Osimertinib in wild-type KRAS mutated cells. Furthermore, since EMT leads to increased cell migration, metastasis, and drug resistance in cancer cells, we analyzed whether knockdown of p120-catenin could inhibit cell migration in a wound-healing assay in H358OR cells. The results indicated that these cells showed slower cell migration in the presence of Osimertinib after p120-catenin knockdown compared to the mock siRNA-treated cells. These results suggest that p120-catenin could be targeted to restore Osimertinib sensitivity by reversing EMT and could potentially positively impact therapeutic outcomes in NSCLC.

Next, we compared p120-catenin and PRMT-1 expression in smokers, quit-smokers, and non-smokers. This is clinically important as cigarette smoking is the major factor driving the pathogenesis and progression of lung cancer. Previous reports have shown that cigarette smoking plays a role in EMT induction [15,16,17]. An earlier report found that lung cancer patients, who were either former or current smokers, had lower levels of E-cadherin and lower overall survival than patients with no smoking history [36]. This report also suggested that smoking-induced EMT is associated with upregulation of LEF-1, a cofactor for β-catenin, and Slug, which both inhibit E-cadherin expression and induce EMT (Figure 8). Another study suggested that cigarette smoke initiates EMT through the Rac1/PI3K/Akt and Rac1/Smad2 signaling pathways and that these pathways are potential targets of therapy [37]. It has previously been shown that the EMT biomarkers PRMT-1 and p120-catenin were abnormally expressed in NSCLC tumors and promoted EMT [38,39,40]. The results of our study demonstrated that high cytoplasmic expression of p120-catenin was mainly observed in smokers compared to quit-smokers and non-smokers. Therefore, p120-catenin could be used as a potential therapeutic target to improve prognosis in NSCLC. These studies show that high expression of PRMT-1 and p120-catenin are associated with Osimertinib resistance. Since p120-catenin may be associated with smoking, it could be used in a clinical setting for NSCLC patients.

Although our results suggest reversal of EMT by upregulation of E-cadherin, a potential limitation of the study is a need to further validate the reversal by exploring the effect on other EMT-related proteins, such as Twist, which is modulated by PRMT-1 [21]. Additionally, the study can be made more impactful if we studied these EMT-proteins in Osimertinib-resistant BA/F3 cells harboring triple mutations (L858R/T790M/C797S) [41]. Overexpression of PRMT-1 and p120-catenin in the parental TKI-sensitive NSCLC cell lines could be an alternative approach to reinforce our current findings. Lastly, in a future study, a comprehensive pathway analysis using RNA-seq or proteomics on EGFR-TKI-resistant cells and tumor samples would help us characterize the modulations of downstream pathways in further detail.

## 5. Conclusions

Overall, this study demonstrated that the EMT biomarkers PRMT-1 and p120-catenin play an important role in activation of the EMT process, increasing the invasive characteristics of NSCLC and, thus, promoting Osimertinib-resistance. We further demonstrated that, by knockdown of p120-catenin, we were able to increase the efficacy of Osimertinib, suggesting that EMT may play a significant role in contributing to Osimertinib resistance. Finally, immunohistochemistry analysis of p120-catenin levels with the smoking status of NSCLC patients suggested that high expression of p120-catenin was mainly observed in smokers as compared to quit-smokers and non-smokers. This data on p120-catenin could potentially help in improving the prognosis of NSCLC patients. Thus, our study on EMT biomarkers opens new opportunities to explore Osimertinib resistance mechanisms and study novel potential therapeutic targets in NSCLC.

## Figures and Tables

**Table 1:** List of qPCR Primers used with sequence information.

Gene	Sequence	Melting Point	DNA Bases
Vimentin	F: TGTCCAAATCGATGTGGATGTTTC	55.7 °C	24
	R: TTGTACCATTCTTCTGCCTCCTG	56.8 °C	23
PRMT-1	F: CCTTCACCTCCCCGTTCTG	57.8 °C	19
	R: CCAGGGCGTGCACGTAGT	60.6 °C	18
p120-catenin	F: CGGCATACGTCATCCCCATT	60.25 °C	20
	R: TCTTCCCTCAGCCCTCAAGT	60.18 °C	20
Kaiso factor	F: GGAAGCAGTGTCTGTTGACT	54.9 °C	21
	R: CCATGCCCTTTCCTCTTTCT	54.9 °C	23
GAPDH	F: ATGACATCAAGAAGGTGGTG	52.4 °C	20
	R: CAGGAAATGAGCTTGACAAA	50.9 °C	20

## Data Availability

Data is contained within the present article and the Supplementary Materials.

## Supplementary Materials

The following supporting information can be downloaded at: https://www.mdpi.com/article/10.3390/cancers15133461/s1, Table S1: Demographics for Smokers, Quit-Smokers and Non-Smokers from whom NSCLC Tumor Tissues were used for Study of PRMT-1/P120-Catenin Expression; Table S2: High and Low Expression of P120-Catenin and the Smoking Status of NSCLC Patients; Table S3: High and Low Expression of PRMT-1 and the Smoking Status of NSCLC Patients; Table S4: Replicates of H358OR Western Blot; Table S5: Replicates of H1975OR Western Blot; Table S6: Replicates of MTT Assay After p120-catenin siRNA Knockdown.
