# Supplementary material for: PRMT-1 and p120-Catenin as EMT Mediators in Osimertinib Resistance in NSCLC
Source: Cancers (Basel). 2023 Jul 1;15(13):3461. doi: 10.3390/cancers15133461 (PMC10340662; doi:10.3390/cancers15133461)
Supplement: Supplementary file 1 [file cancers-15-03461-s001.zip › Formatted Westerns.pdf]

WESTERN BLOTS FOR FIGURE 2. Blue arrow indicates known approximate protein molecular weight.

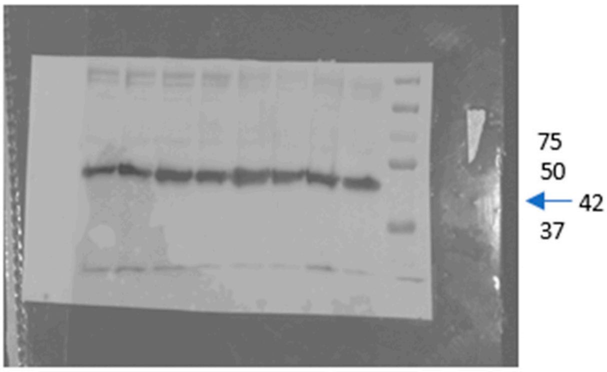

H1975OR β-actin

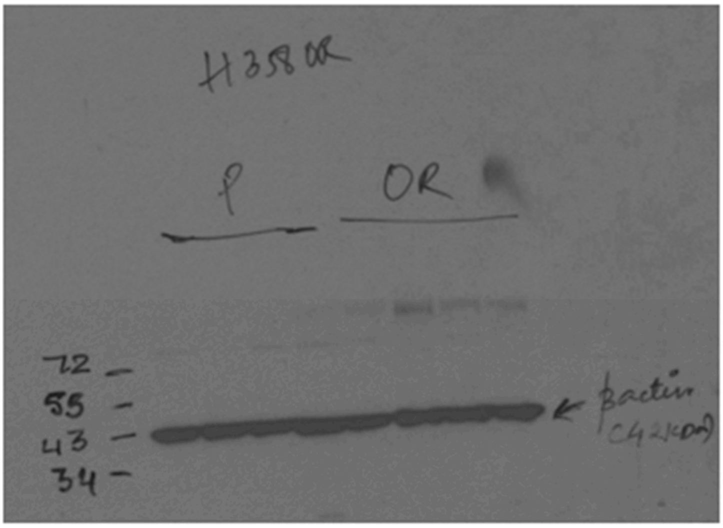

H358OR β-actin

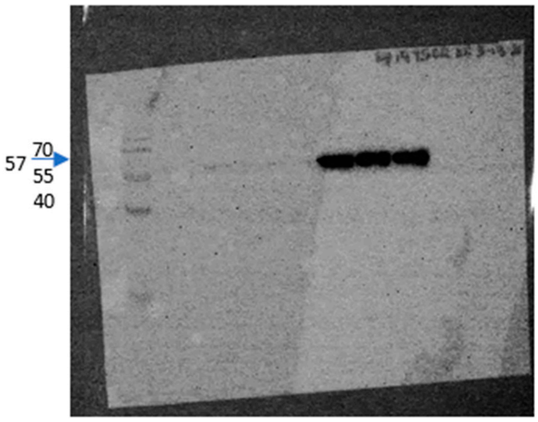

H1975OR Vimentin

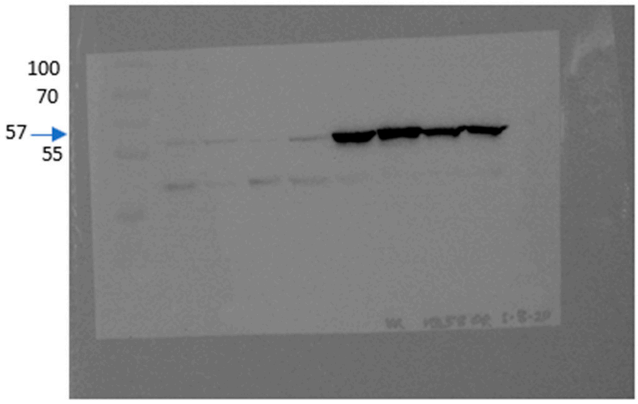

H358OR Vimentin

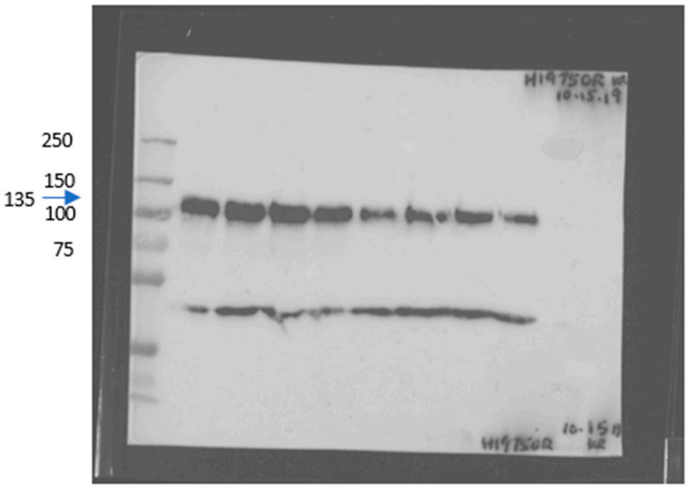

H1975OR E-cadherin

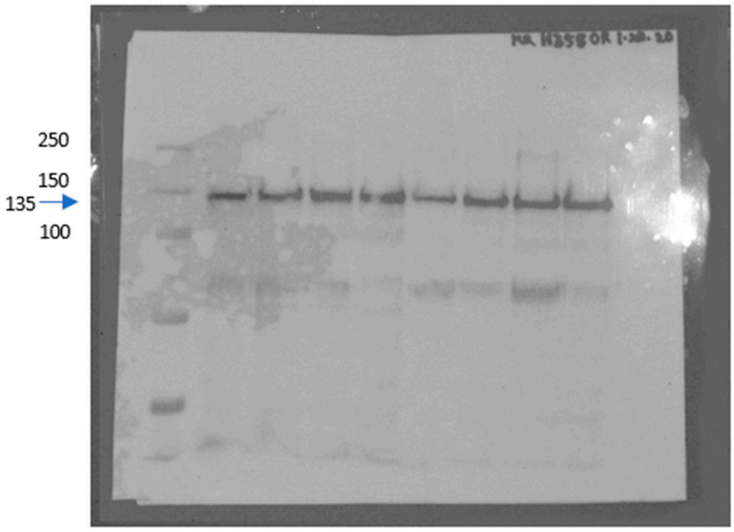

H358OR E-cadherin

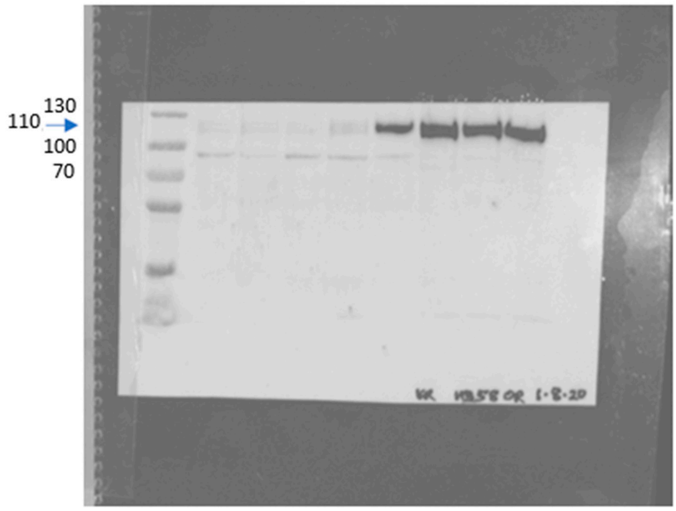

H358OR p120-catenin

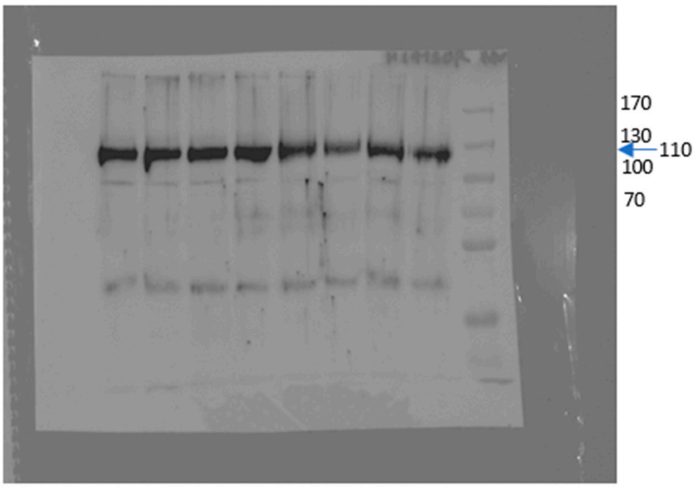

H1975OR p120-catenin

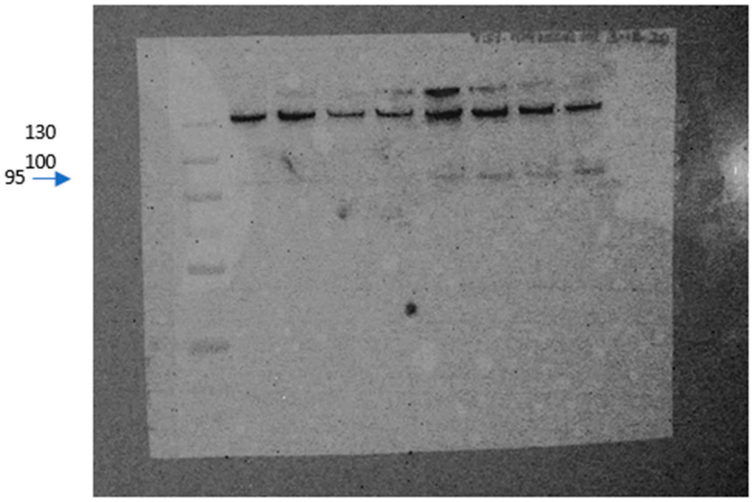

H1975OR Kaiso Factor

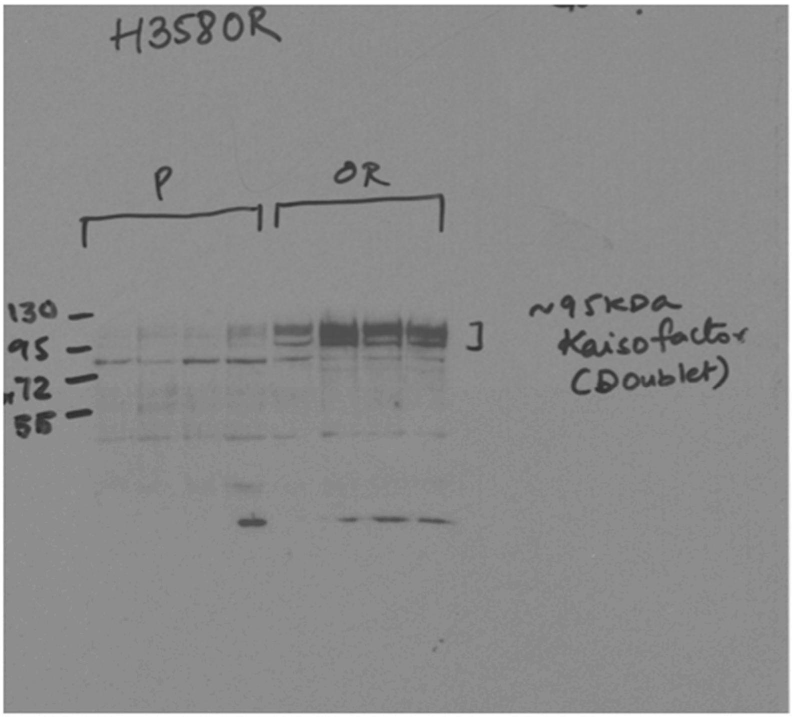

H358OR Kaiso Factor

WESTERN BLOTS FOR FIGURE 5. Blue arrow indicates known approximate protein molecular weight.

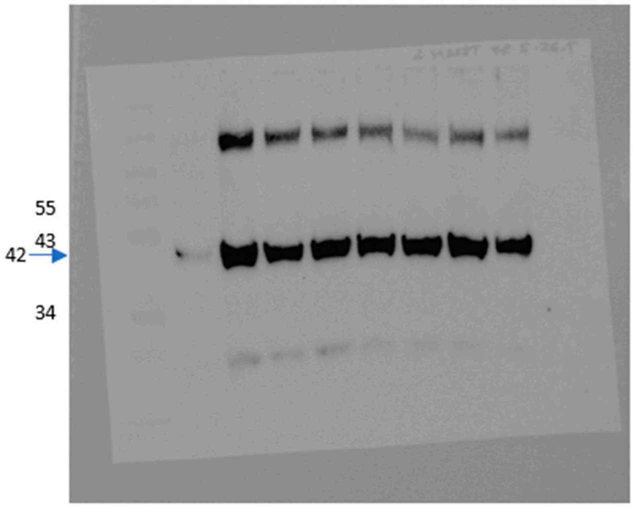

β-actin

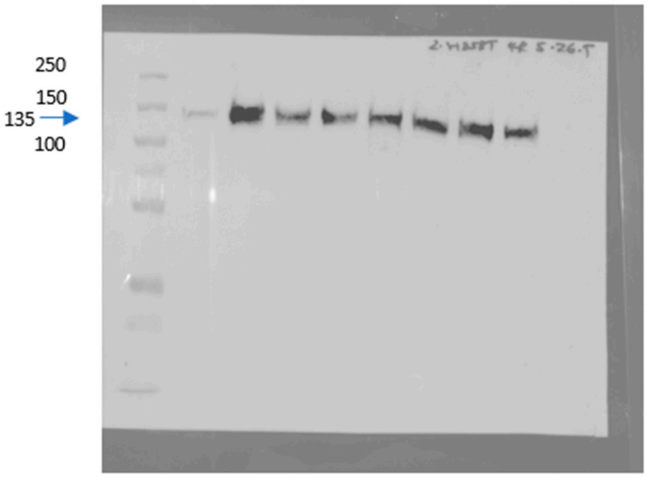

E-cadherin

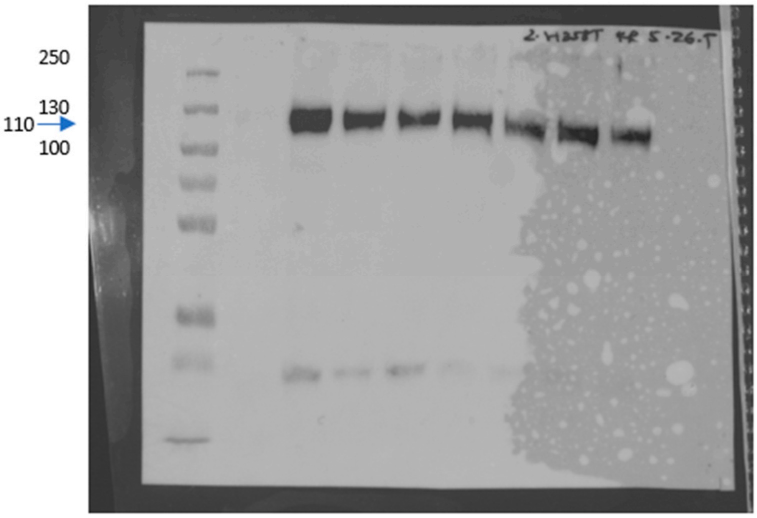

p120-catenin
