# Supplementary material for: PRMT-1 and p120-Catenin as EMT Mediators in Osimertinib Resistance in NSCLC
Source: Cancers (Basel). 2023 Jul 1;15(13):3461. doi: 10.3390/cancers15133461 (PMC10340662; doi:10.3390/cancers15133461)

Supplementary Materials

TABLE S1. DEMOGRAPHICS FOR SMOKERS, QUIT SMOKERS AND NON-SMOKERS FROM WHOM NSCLC TUMOR TISSUES WERE USED FOR STUDY OF PRMT-1/p120-CATENIN EXPRESSION. Demographics for the 22 quit smokers, 20 smokers, and 21 non-smokers from whom NSCLC Tumor Tissues were used for the study of PRMT-1, and p120-catenin expression. The demographic table shows the sex, race and age of the patients chosen in the study. They all were Caucasian and African Americans and ranged in age from 40 to 80 years old. The de-identified tumor sections used for this study have been taken from lung cancer patients prior to any treatment.

Type	Sex		Race		Age		
	Male	Female	Caucasian	African American	40-59 years old	60-79 years old	Above 80 years old
Quit Smokers	9	13	21	1	5	16	1
Smokers	11	9	20	0	5	3	2
Non-smokers	7	14	19	2	4	16	1

TABLE S2. HIGH AND LOW EXPRESSION OF p120-CATENIN AND THE SMOKING STATUS OF NSCLC PATIENTS. Analysis was performed for high and low expression of p120-catenin with the smoking status of NSCLC patients. Statistical analysis was performed using Fisher's exact Test for p120-catenin expression and smoking status of the patients. The p-value was calculated using the Fisher's exact Test with p<0.05 as the significance value.

p120-catenin Expression	Smoking status of NSCLC Patients		
	Frequency		
	Smoker	Quit Smoker	Non-Smoker
High Expression	6	0	1
Low Expression	12	21	17
p-value	0.002		

TABLE S3. HIGH AND LOW EXPRESSION OF PRMT-1 AND THE SMOKING STATUS OF NSCLC PATIENTS. Analysis was performed for high and low expression of PRMT-1 with the smoking status of NSCLC patients. Statistical analysis was performed using Fisher's exact Test for PRMT-1 expression and smoking status of the patients. The p-value was calculated using the Fisher's exact Test with p<0.05 as significant value.

PRMT-1 Expression	Smoking status of NSCLC Patients		
	Frequency		
	Smoker	Quit Smoker	Non-Smoker
High Expression	9	8	10
Low Expression	9	14	10
p-value	0.679		

TABLE S4: REPLICATES OF H358OR WESTERN BLOT. Replicates of Western Blot experiments comparing fold change of EMT-related proteins in H358 parental cells to H358OR (Osimertinib Resistant). Statistical analysis was performed using 2 tailed t-test with p<0.05 as significant value.

E-cadherin	Kaiso factor	Vimentin	p120-catenin
-3.03734	7.538606	24.15785	13.42666
-1.42153	13.96234	45.75979	13.28758
-1.50638	13.53779	37.71268	10.67878
-1.16486	17.70285083	26.80926	18.14814

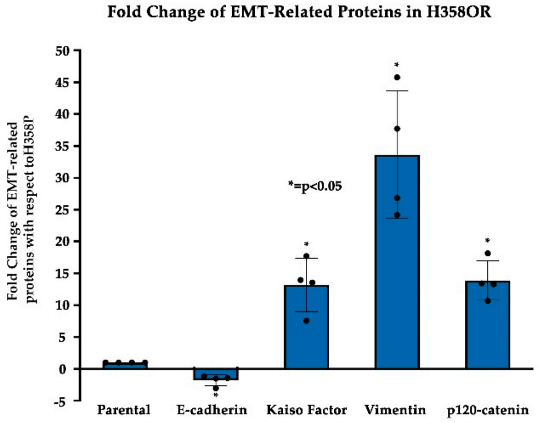

TABLE S5: REPLICATES OF H1975OR WESTERN BLOT. Replicates of Western Blot experiments comparing fold change of EMT-related proteins in H1975 parental cells to H1975OR (Osimertinib Resistant). Statistical analysis was performed using 2 tailed t-test with p<0.05 as significant value.

E-cadherin	Kaiso factor	Vimentin	p120-catenin
-1.65236	4.255447	20.69061	1.490924
-1.44801	5.814773	41.98547	2.168197
-0.99142	4.563008	73.24499	2.163952
-0.937100	2.828507	55.02873	1.272287

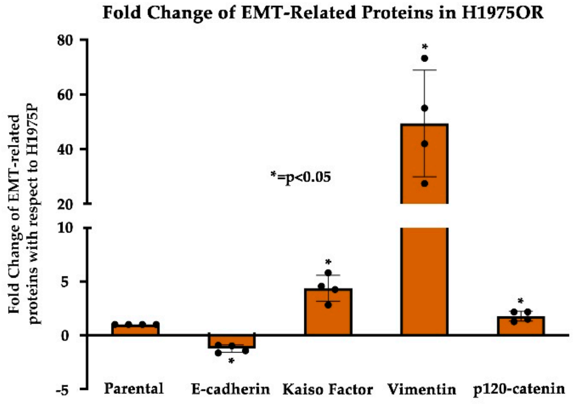

TABLE S6. REPLICATES OF MTT ASSAY AFTER p120-CATENIN siRNA KNOCKDOWN. Replicates of MTT assay were performed on H358OR cells after siRNA mediated knockdown of p120-catenin. Statistical analysis was performed using 2-tailed t-test to compare the combination treatment (p120siRNA and Osimertinib) with mock and with mock in combination with Osimertinib. The p-value was calculated using $p<0.05$ as significant value.

Mock	Mock+OR	p120 siRNA	p120si RNA+OR
100	89.28287	86.4940239	30.87649
100	89.28287	96.29482072	64.50199
100	83.78486	95.17928287	30.95618

Relative Cell Viability in H358OR after siRNA mediated knockdown of p120-catenin as determined by MTT cell viability assay

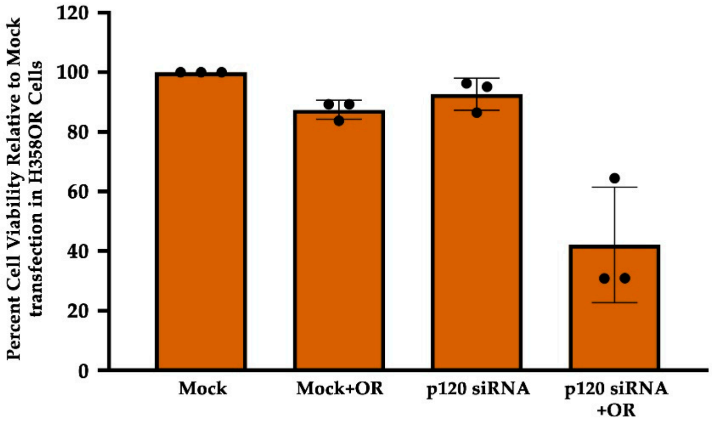

Supplement: Supplementary file 1 [file cancers-15-03461-s001.zip › Racherla et. al. Supplementary data.pdf]
